# Olaparib and ionizing radiation trigger a cooperative DNA-damage repair response that is impaired by depletion of the VRK1 chromatin kinase

**DOI:** 10.1186/s13046-019-1204-1

**Published:** 2019-05-17

**Authors:** Ignacio Campillo-Marcos, Pedro A. Lazo

**Affiliations:** 1 Experimental Therapeutics and Traslational Oncology Program, Instituto de Biología Molecular y Celular del Cáncer, Consejo Superior de Investigaciones Científicas (CSIC)-Universidad de Salamanca, 37007 Salamanca, Spain; 2 Instituto de Investigación Biomédica de Salamanca (IBSAL), Hospital Universitario de Salamanca, 37007 Salamanca, Spain

**Keywords:** VRK1, Vaccinia-related kinase 1, Olaparib, Ionizing radiation, H2AX, NBS1, 53BP1, Histone H4, DNA repair

## Abstract

**Background:**

The VRK1 chromatin kinase regulates the organization of locally altered chromatin induced by DNA damage. The combination of ionizing radiation with inhibitors of DNA damage responses increases the accumulation of DNA damage in cancer cells, which facilitates their antitumor effect, a process regulated by VRK1.

**Methods:**

Tumor cell lines with different genetic backgrounds were treated with olaparib to determine their effect on the activation of DNA repair pathways induced by ionizing radiation. The effect of combining olaparib with depletion of the chromatin kinase VRK1 was studied in the context of double-strand breaks repair pathway after treatment with ionizing radiation. The initiation and progression of DDR were studied by specific histone acetylation, as a marker of local chromatin relaxation, and formation of γH2AX and 53BP1 foci.

**Results:**

In this work, we have studied the effect that VRK1 by itself or in collaboration with olaparib, an inhibitor of PARP, has on the DNA oxidative damage induced by irradiation in order to identify its potential as a new drug target. The combination of olaparib and ionizing radiation increases DNA damage permitting a significant reduction of their respective doses to achieve a similar amount of DNA damage detected by γH2AX and 53BP1 foci. Different treatment combinations of olaparib and ionizing radiation permitted to reach the maximum level of DNA damage at lower doses of both treatments. Furthermore, we have studied the effect that depletion of the VRK1 chromatin kinase, a regulator of DDR, has on this response. VRK1 knockdown impaired all steps in the DDR induced by these treatments, which were detected by a reduction of sequential markers such as H4K16 ac, γH2AX, NBS1 and 53BP1. Moreover, this effect of VRK1 is independent of *TP53* or *ATM*, two genes frequently mutated in cancer.

**Conclusion:**

The protective DNA damage response induced by ionizing radiation is impaired by the combination of olaparib with depletion of VRK1, and can be used to reduce doses of radiation and their associated toxicity. Proteins implicated in DNA damage responses are suitable targets for development of new therapeutic strategies and their combination can be an alternative form of synthetic lethality.

**Electronic supplementary material:**

The online version of this article (10.1186/s13046-019-1204-1) contains supplementary material, which is available to authorized users.

## Background

Therapies targeting to DNA damage response (DDR) pathways are becoming a model for identification of suitable novel combination therapies, which include inhibition of DNA repair mechanisms and chromatin regulation [1, 2]. One mainstay of cancer treatment is based on the generation of catastrophic DNA damage in tumor cells, particularly those that directly damage DNA, which include ionizing radiation (IR), among others**.** IR generates reactive oxygen species that attacks DNA mostly causing single-strand, and if not repaired, double-strand breaks. Therefore, interference with DNA repair (DDR) mechanisms can facilitate and increase the amount of DNA damage in cells. In this context, new drugs have been developed for targeting DNA repair processes and for use in tumors with deficiency in DDR pathways, generally linked to *ATM* and *BRCA* mutations.

DNA damage causes a local distortion of chromatin, which triggers several sequential reactions in order to start the appropriate DNA damage response (DDR) [3, 4]. These sequential steps range from a local chromatin relaxation and remodeling, and protection of DNA at damaged sites, to the recognition of the type of damage and the activation of its specific DDR pathway. Among these early events is histone acetylation, which is associated with a local chromatin relaxation that facilitates accessibility to the proteins in the DDR sequential steps, which include phosphorylation of H2AX and recruitment of DNA repair proteins, such as NBS1, NBS1 and 53BP1, implicated in Non-homologous end-joining (NHEJ), a key DDR pathway in resting cells.

Olaparib is an inhibitor of poly-ADP ribose polymerase (PARP), a component of the base-excision repair (BER), that is involved in the repair of DNA damage caused by oxidative stress [5, 6]. Because of that, PARP has become an important therapeutic target [7]. Olaparib inhibits end-joining mediated by PARP [8] and sensitizes cells to DNA damage induced by ionizing radiation [9–12]. The interference of this repair pathway with olaparib facilitates that single-strand breaks become double-strand breaks, promoting the accumulation of DNA damage and the subsequent cell death [13]. Therefore, PARP inhibitors, such as olaparib, confer cytotoxicity in response to high levels of reactive oxygen species [14] and are used in the treatment of tumors lacking ATM [15, 16] or BRCA1 [17, 18], which are deficient in DDR and more sensitive to genotoxic treatment.

Targeting DDR is a form of cancer treatment [1]. The sequential steps in DDR require a coordination that is mediated by the chromatin kinase VRK1 [19]. The VRK1 kinase appeared late in evolution in higher eukaryotes and is regulated independently of the type of DNA damage [20]. The chromatin and nucleosomal-kinase VRK1 [19, 21] is a Ser-Thr kinase associated to processes that require a dynamic chromatin remodeling, including cell proliferation [22] and DNA damage responses [19, 20, 23, 24]. VRK1 participates in these events by its direct involvement in specific repair processes at different sequential stages. Initially, VRK1 depletion impairs chromatin remodeling by regulating histone acetylation [24, 25], required for relaxation of chromatin at sites of DNA breaks [24]. Later in the response, VRK1 depletion also impairs specific steps in pathway involved in DNA repair and interferes with the formation of γH2AX [24], NBS1 [23] or 54BP1 [20] foci. Thus, the combination of VRK1 with ionizing radiation or doxorubicin results in an increased sensitivity to these commonly used treatments [26]. In this context, high levels of VRK1 confers resistance to doxorubicin treatment [26]. Furthermore, high VRK1 levels are also associated to very poor prognosis in many types of carcinomas with different origin and genetic background [27–30].

It is becoming very evident that combinations of treatments might result in improvement of cancer treatment based on synthetic lethality, and, at the same time, require the use of a lower drug dosage with respect to their individual use, which can also have the benefit of a reduced toxicity. In this work we have studied the effect that VRK1 depletion has on the cellular response to olaparib, a drug which is currently used to sensitize tumor cell to ionizing radiation, and facilitates tumor elimination in cells with altered DNA repair pathways, such as those with *BRCA1* [17, 18] or *ATM* [15, 16] mutations. Based on this, we postulated that kinases that regulate sequential steps in DDR mechanisms are potential drug targets that can sensitize tumor cells to chemotherapy and IR because of an impaired DDR, which will result in a loss of tumor cell viability, particularly if these tumors have mutations in *ATM*, *BRCA1* or *TP53* genes. Therefore, the identification of new drug combinations targeting different DDR pathways will be a suitable form of treatment and lead to a reduction in doses of the current therapies, which are frequently very toxic for cancer patients.

## Materials and methods

### Reagents and inhibitors

Olaparib was from LC Laboratories (Woburn, MA, USA), and KU55933, from Tocris Bioscience (Bristol, UK). All other reagents were from Sigma-Aldrich-Merck (Darmstadt, Germany).

### Cell lines and culture

MDA-MB-231 (triple negative), A549 (*TP53*+/+) and HT144 (*ATM*−/−) cell lines were obtained from the ATCC and grown as recommended by the supplier in DMEM supplemented with antibiotics, 10% FBS and 5 mM glutamine. H1299 (*TP53*−/−) cell line was grown in RPMI medium supplemented with antibiotics, 10% FBS and 5 mM glutamine. All cell lines are mycoplasma free.

### RNA interference

The depletion of VRK1 by siRNA has been previously reported for A549 [20, 23], H1299 [20, 23], MDA-MB-231 [26] and HT144 [23, 24] cell lines. Specific VRK1 knockdown was performed using siVRK1–02 from DHARMACON RNA Technologies. The target sequences of this siVRK1–02 is the following one (5′ to 3′): CAAGGAACCUGGUGUUGAA. The “ON-TARGETplus siCONTROL Non-targeting siRNA” from DHARMACON was used as negative control (siCtrl) [31]. Briefly, cells were transfected with the indicated siRNA at a concentration of 20 nM using either Lipofectamine 2000 (Invitrogen) or Lipotransfectin (Nivorlab), following manufacturer’s instructions [25, 31, 32].

### DNA damage

DNA damage was induced by ionizing radiation with 0.5, 1 or 3 Gy using a Gammacell 1000 Elite irradiator (Theratronics, Ottawa, Canada) with a ^137^Cs source, and exposure to different concentrations of Olaparib (PARP inhibitor, LC Laboratories; Woburn, MA, USA), or their combinations, as indicated in the corresponding experiments. The doses of olaparib were added to the culture for twenty-four hours before irradiation.

### Cell lysates and acid extraction of histones

Protein extracts were performed using two different lysis buffers, “Suave” and RIPA, depending on the cell line. The “Suave” lysis buffer was composed by 50 mM Tris–HCl (pH 8.0), 1 mM EDTA, 150 mM NaCl, and 1% Triton X-100, while the RIPA one contained 150 mM NaCl, 1.5 mM MgCl_2_, 10 mM NaF, 4 mM EDTA, 50 mM Hepes, 1% Triton X-100, 0.1% SDS, and 10% glycerol. In both cases, these buffers also contained phosphatases inhibitors (1 mM NaF and 1 mM sodium orthovanadate) and proteases inhibitors (1 mM PMSF, 10 μg/mL aprotinin, and 10 μg/mL leupeptin), which were added before starting the lysis itself. Acidic extracts of histones were performed as previously reported [33]. Protein extracts were quantified using the Bradford protein assay (Bio-Rad; Hercules, CA) in order to load the same amount of these extracts in each well of the polyacrylamide gel, and boiled at 100 °C in Laemmli buffer for 5 min [32, 34].

### Antibodies

All the antibodies are listed in Table 1. These antibodies, used in immunoblots and/or immunofluorescence assays, were diluted in TBS-0.1% Tween20 or PBS-1% BSA, respectively.

**Table 1:** List of antibodies and conditions used in this work

Antibody		Dilution (WB/IF)	Clone and/or reference	Supplier
53BP1	Rabbit polyclonal	-; 1/200	H300, sc-22,760	Santa Cruz Biotechnology
53BP1	Rabbit polyclonal	-; 1/200	NB100–304	Novus Biologicals
Histone H4-K16 ac	Rabbit monoclonal	1/500; 1/400	ab109463	Abcam
Nbs1	Mouse monoclonal	-; 1/200	611,871	BD Biosciences
VRK1	Mouse monoclonal	1/1000; 1/200	1B5	Own production [74]
VRK1	Rabbit polyclonal	1/1000;-	VC	Own production [74]
VRK1 (N-term)	Rabbit polyclonal	-; 1/200	HPA000660	Sigma-Aldrich
β-actin	Mouse monoclonal	1/2000; −	AC15/A5441	Sigma-Aldrich
γH2AX	Mouse monoclonal	-; 1/200	Clone JBW301; 05–636	Millipore
Anti-mouse IgG (WB)	Goat Anti-Mouse IgG, DyLight 680 (red)	1/10000; −	35,518	Thermo Scientific
Anti-rabbit IgG (WB)	Goat Anti-Rabbit IgG, DyLight 800 (green)	1/10000; −	35,571	Thermo Scientific
Goat anti-Mouse IgG (IF)	Goat anti-Mouse IgG linked to Cy3 (red)	-; 1/1000	115–165-146	Jackson ImmunoResearch; West Grove, PA, USA
Goat anti-rabbit IgG (IF)	Goat anti-rabbit IgG linked to Cy2 (green)	-; 1/1000	111–225-144	Jackson ImmunoResearch; West Grove, PA, USA

### SDS-page electrophoresis and western blot analysis

Proteins were fractionated by SDS-Page vertical electrophoresis and transferred to Immobilon-FL membranes (Millipore). The membranes were blocked with TBS-T buffer (25 mM Tris-HCl (pH 8), 50 mM NaCl, 2.5 mM KCl, 0.1% Tween-20) and 5% nonfat dry milk, for 1 h at room temperature. Blocked membranes were incubated with the primary antibody (listed in Table 1) for an additional 1 h at room temperature or overnight at 4 °C, followed by extensive washing in TBS-T buffer (3 × 10 min). Next, membranes were incubated with the corresponding secondary antibodies (Table 1) for 1 h in darkness, followed by three washes with TBS-T buffer. Finally, membrane signals were detected using the LI-COR Odyssey Infrared Imaging System (LI-COR Biosciences; Lincoln, NE, USA).

### Immunofluorescence and confocal microscopy

Cells were plated on 60 mm dishes with several coverslips, two for each antibody to be used. Cells on coverslips were fixed with 3% paraformaldehyde for 30 min, and treated with a solution of glycine 200 mM for 15 min at room temperature. Next, these cells were permeabilized with 0.2% Triton X-100 solution in PBS for 30 min and blocked with 1% BSA in PBS for 30 min at room temperature or overnight at 4 °C. For the simultaneous detection of two proteins, each coverslip was sequentially incubated with the two primary antibodies, followed by three washes for 10 min in PBS after each antibody. The incubation with the corresponding secondary antibodies, labeled with Cy2 or Cy3 (Table 1 section), was performed in darkness for 1 h at room temperature. After three washes for 10 min in PBS, nuclei were stained with DAPI (4′, 6′-diamidino-2-phenylindole) (Sigma) 1:1000 in PBS for 15 min at room temperature. Finally, cells were washed three times for 10 min in PBS and slides were mounted with Mowiol (Calbiochem-Merck, Darmstadt, Germany). Fluorescent images were acquired with a LEICA SP5 DMI-6000B confocal microscope (Leica), using the following lasers: Argon (488 nm), DPSS (561 nm) and UV Diode (405 nm). These images were captured with a 63.0x lens zoomed in 1.5–3× with a 1024 × 1024 frame and 600 Hz scanning speed. Scanner settings were maintained constant throughout all samples examined: pinhole (95.6 μm), lasers intensity and photomultipliers gain and offset. Afterwards, images were analyzed with ImageJ software (https://imagej.nih.gov/ij/).

### Statistical analysis

All these statistical analysis were performed using IBM SPSS 25 statistics package. Quantitative experiments were repeated, at least, three times and statistical significance was analyzed using Bonferroni adjusted t-tests (post hoc parametric test) or Dunn’s multiple comparison tests (post hoc non parametric test), depending on whether all samples were adjusted to a normal distribution or not, respectively. In both cases, the level of significance was 0.05 (*, *p* < 0.05; **, *p* < 0.01; and ***, *p* < 0.001). All results are represented as box plots, in which the box ranges from the end of first quartile to the third quartile, the line cutting the box represents the median, and the whiskers extend to the minimum or maximum observations [35].

## Results

### Olaparib sensitizes cells to DNA damage induced by ionizing radiation and is independent of p53

**Fig. 1:**
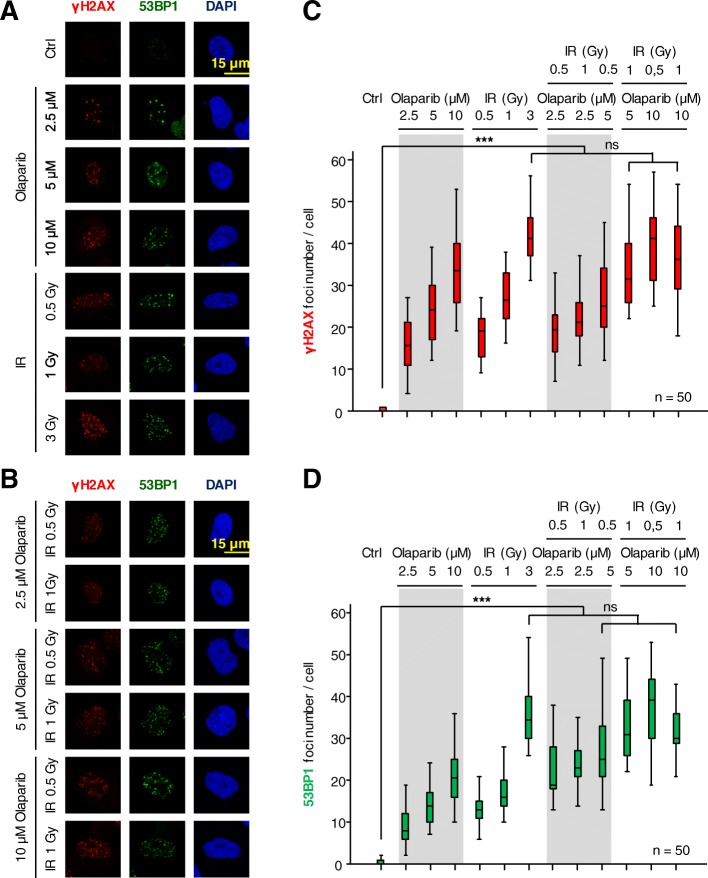
Effect of combinations of olaparib and ionizing radiation on the formation of γH2AX and 53BP1 foci in response to DNA damage in A549 cells. **a**. Effect of different doses of either olaparib or ionizing radiation on the formation of γH2AX and 53BP1 foci in response to DNA damage. **b**. Effect of combinations of olaparib and ionizing radiation on the formation of γH2AX and 53BP1 foci. **c**. Quantification of the effect of olaparib and IR by themselves or in combination on the number of γH2AX foci. **d**. Quantification of the effect of olaparib and IR, by themselves or in combination, on the number of 53BP1 foci. ns: not significant, *** *p* < 0.001. The images show the detail of the subnuclear protein detected. The quantifications were performed using fifty cells from different fields of the experiments (usually between seven and ten were required). The field images and the image selected for presentation in the main figure are indicated in Additional file 1: Figure S1

Initially, we studied the effect of several doses of IR, olaparib or their combinations using two markers of DNA damage response (DDR). Phosphorylated histone H2AX in Ser139 (γH2AX) was used as an early indicator of DNA damage [36], and 53BP1 as marker of DNA double-strand break repair by the NHEJ pathway [37], which are key regulators at different stages of the repair process [38]. These foci are indirect markers of the extent of DNA damage and represent stalled repair in DNA damage areas. The effect of different doses of olaparib and IR was determined in lung cancer A549 (*TP53*+/+) cells (Fig. 1a), as well as their combination (Fig. 1b). These results showed that a combination of lower doses of olaparib and IR on the formation of γH2AX and 53BP1 foci (Fig. 1b) was required to reach a similar effect to those caused by higher doses of either IR or olaparib individually used. The quantifications of foci are shown for γH2AX (Fig. 1c) and 53BP1 (Fig. 1d). These γH2AX and 53BP1 foci indicate the aggregation of these proteins induced by DNA damage, without an effect on their protein levels in the short observation times. The combination of lower doses of olaparib (5 μM) and IR (1 Gy) permitted to reach the same number of γH2AX and 53BP1 foci that were achieved with the highest dose of radiation (3Gy). This represented a very significant reduction in the dose of radiation used, from 3 to 1 Gy, to reach a similar amount of DNA damage.

The p53 tumor suppressor is a mediator of multiple responses to different types of cellular stress, including DNA damage, which consequently triggers cell cycle arrest or apoptosis by a complex signaling network [39]. To rule out whether the effect of olaparib on sensitization to IR is mediated by a p53**-**dependent mechanism, a similar experiment was performed in H1299 (*TP53*−/−) lung cancer cells (Additional file 2: Figure S2). The formation of γH2AX and 53BP1 foci in response to olaparib or IR, and their combination, led to the same conclusion and they are independent of p53. The combination of olaparib and IR permitted a reduction in dose of olaparib, from 10 to 5 μM, and IR, from 3 to 1 Gy, to reach a similar effect on the number of DNA damaged sites detected by 53BP1 foci. A similar result was obtained using the breast triple-negative cancer cell line MDA-MB-231 (Additional file 3: Figure S3). Therefore, it can be concluded that the combination of lower doses of olaparib and IR generates a maximum level of DNA damage, as detected by the formation of repair foci, which could only be reached by using higher doses of olaparib or IR in the absence of their combination.

### VRK1 depletion impairs the acetylation of K16 in histone H4 induced by olaparib and IR

**Fig. 2:**
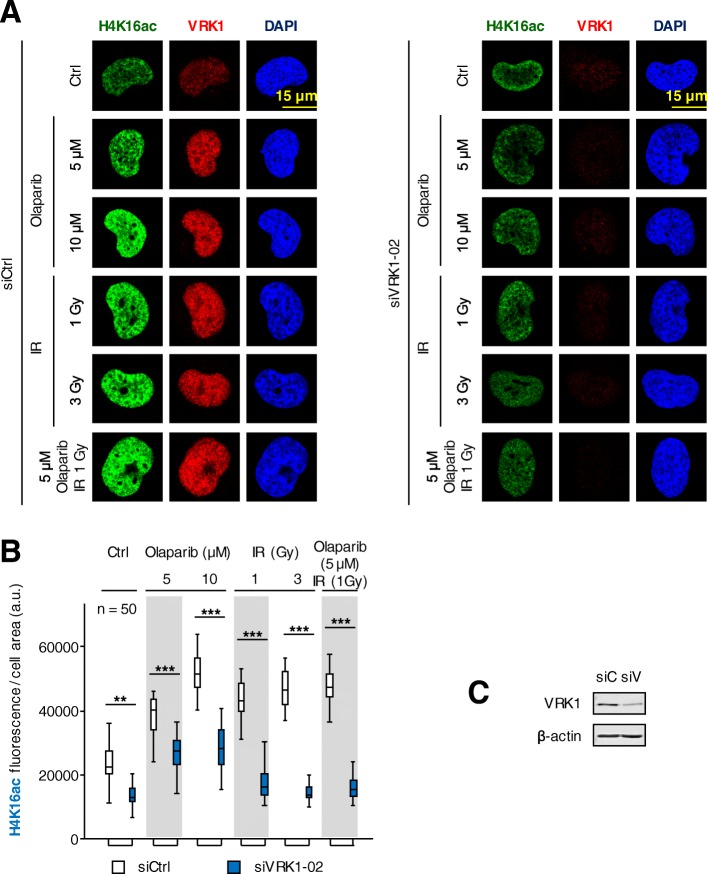
Effect of VRK1 depletion on the nuclear fluorescence associated to the acetylation of histone H4 in lysine 16 (H4K16ac ) induced by olaparib, IR or their combination in H1299 (*TP53*−/−) cells deprived (0.5%) of serum. **a** left. Effect of si-Control (siCtrl/siC) on H1299 cells treated with different doses of olaparib, IR or their combination on nuclear H4K16 ac fluorescence. **a** right. Effect of si-VRK1 (siVRK1/siV) on H1299 cells treated with different doses of olaparib, IR or their combination on the acetylation of histone H4 in lysine 16. **b**. Quantification of the effect of VRK1 depletion on the increase of nuclear H4K16ac fluorescence induced by DNA damage. **c**. The immunoblot shows the effect of VRK1 depletion on its protein level. siC: siControl, siV: siVRK1–02. ns: not significant, ** *p* < 0.01, *** *p* < 0.001. The images show the detail of the subnuclear protein detected. The quantifications were performed using fifty cells from different fields of the experiments (usually between seven and ten were required). The images selected for presentation in the main figure are indicated in Additional file 4: Figure S4. Similar results were obtained in A549 cells (*TP53*+/+) (Additional file 5: Figure S5) in serum (0.5%) deprived cells

The first event that occurs following DNA damage is an aberrant local disruption of chromatin that needs to be reorganized in order to start and permit the appropriate DDR depending on the type of DNA damage. This initial chromatin relaxation is associated with histone acetylation [40], which can be monitored by following the acetylation of histone H4 in K16 [41–43]. This specific histone acetylation facilitates the access to DNA repair proteins at damaged sites, and its loss is associated with a defective DNA repair [44]. Therefore, we studied the effect on H4K16 acetylation in response to treatment with olaparib, ionizing radiation or their combination as well as VRK1 depletion, in different cell types. A maximal effect was achieved by the combination of olaparib with IR on H4K16 acetylation fluorescence in H1299 (*TP53*−/−) (Fig. 2) and A549 (Additional file 5: Figure S5) lung cancer cell lines deprived (0.5%) of serum. The combination of olaparib (5 μM) with 1Gy achieved the same effect on nuclear H4K16 ac fluorescence as 3Gy only, and depletion of VRK1 impaired the level of H4K16ac associated with chromatin relaxation induced by DNA damage. This observation suggested that VRK1 depletion comprised the correct progression of later stages in the DDR process. In addition, depletion of VRK1 caused a cell cycle arrest and prevents cell proliferation [45–47] and chromatin condensation in mitosis [48, 49]. Consequently, cells will not be able to divide or repair its damaged DNA.

### VRK1 depletion interferes with the DNA damage response mediated by NBS1

**Fig. 3:**
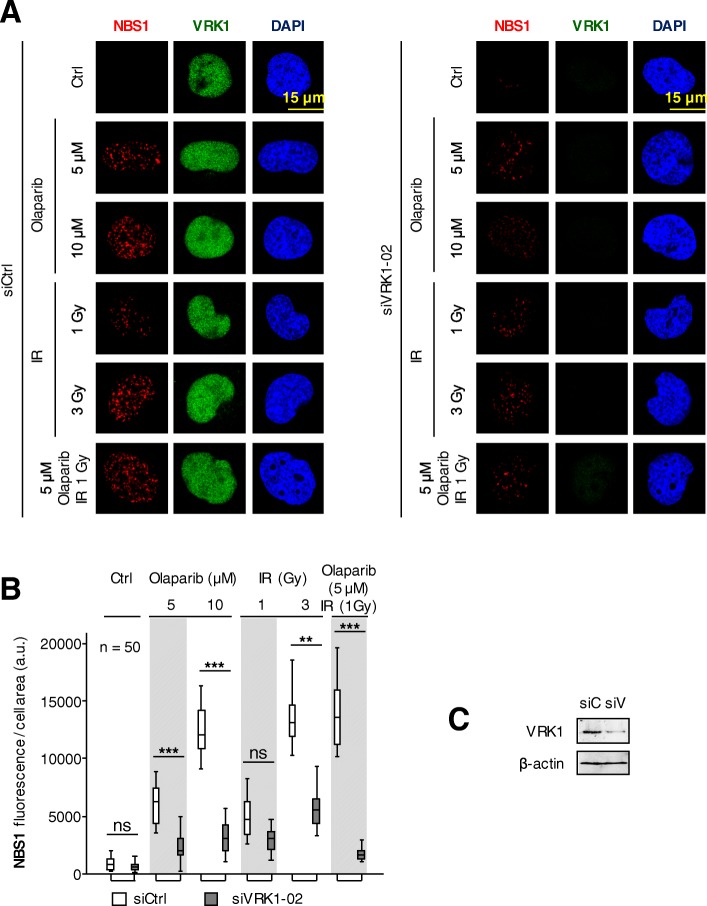
Effect of VRK1 depletion on nuclear NBS1 fluorescence induced by olaparib, IR or their combination in H1299 (*TP53*3−/−) cells arrested by the absence of serum. A left. Effect of si-Control on H1299 cells treated with different doses of olaparib, IR or their combination on the NBS1 fluorescence. **a** right. Effect of si-VRK1 on H1299 (*TP53*−/−) cells treated with different doses of olaparib, IR or their combination on the accumulation of NBS1 foci in nuclei. **b**. Quantification of the effect of VRK1 depletion on the increase of nuclear NBS1 fluorescence by aggregation of this protein induced by DNA damage. Similar results were obtained in A549 (*TP53*wt) (Additional file 7: Figure S7), and HT144 (*ATM*−/−) cells (Additional file 8: Figure S8) in the absence of serum (0.5%). **c.** The immunoblot shows the effect of VRK1 depletion on its protein level. siC: siControl, siV: siVRK1–02. ns: not significant, ** *p* < 0.01, *** p < 0.001

The phosphorylation and activation of NBS1 is an early step in the DDR response [50, 51], and VRK1 regulates this phosphorylation [23]. Therefore, we studied the effect of VRK1 depletion on the NBS1 response to treatment with olaparib, ionizing radiation or their combination in several cell lines with different genetic alterations. The combination of olaparib (5 μM) with 1Gy achieved the same effect on NBS1 nuclear fluorescence as 3Gy only (Fig. 3, control), as with H4K16ac  acetylation. The depletion of VRK1 impaired the DNA damage response mediated by NBS1 in H1299 (*TP53*−/−) (Fig. 3, Additional file 6: Figure S6), A549 (Additional file 7: Figure S7), and HT144 (*ATM−/−*) cells (Additional file 8: Figure S8) deprived (0.5%) of serum. These data indicated that the combination of olaparib with IR permits a reduction in dose of IR and olaparib, and is independent of ATM or p53.

### VRK1 depletion impairs the DNA damage response triggered by olaparib and IR

**Fig. 4:**
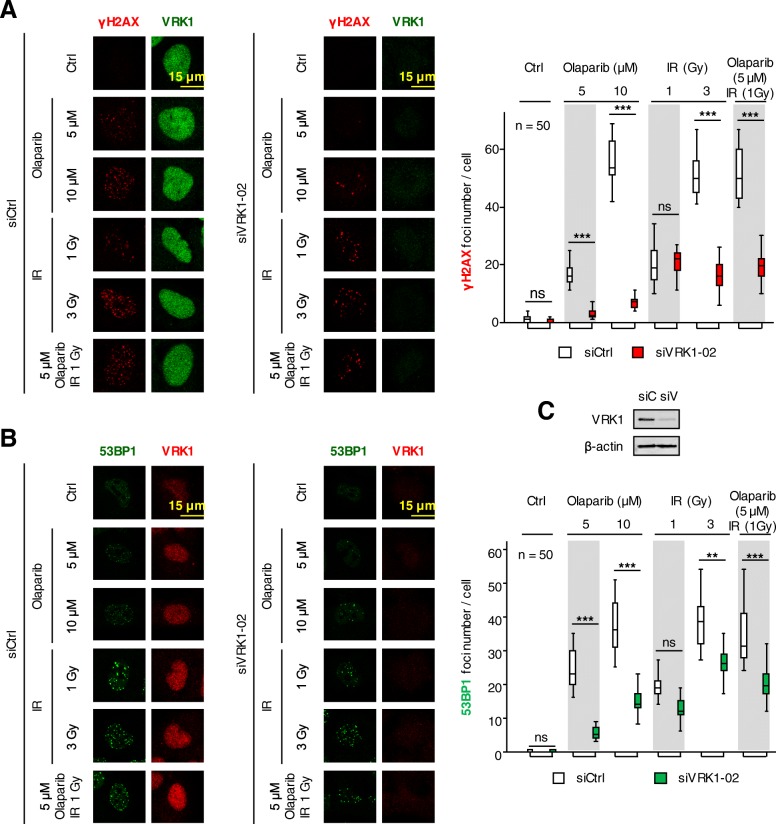
Effect of VRK1 depletion on the formation of γH2AX and 53BP1 foci induced by olaparib, IR or their combination in A549 cells. **a**. Effect of si-Control on A549 cells treated with different doses of olaparib, IR or their combination on the formation of γH2AX foci. **b**. Effect of si-VRK1 on A549 cells treated with different doses of olaparib, IR or their combination on the formation of 53BP1 foci. **c**. Detection of VRK1 depletion in immunoblot. siC: siControl, siV: siVRK1–02. ns: not significant, ** *p* < 0.01, *** *p* < 0.001

DNA damage triggers a local reorganization of chromatin, and olaparib or IR, which interferes with the repair of oxidative DNA damage and causes oxidative stress, respectively, can induce this damage. DNA damage triggers a local chromatin reorganization regulated by kinases among which is VRK1 [19]. This chromatin kinase is necessary for chromatin relaxation and the correct sequential phosphorylation and activation of histone H2AX [24], NBS1 [23] and 53BP1 [20, 26] in resting cells, in which double-strand breaks are repaired by the Non-homologous end-joining pathway [20, 23, 24]. Therefore, we hypothesized that depletion of endogenous VRK1 can interfere with the response to olaparib and prevented the sequential DNA repair steps. In A549 cells, VRK1 depletion by siRNA resulted in a defective DDR that was detected as a loss of γH2AX (Fig. 4a), and 53BP1 (Fig. 4b) foci formed in response to DNA damage. These effects are due to the inability of VRK1-depleted cells to assemble DNA repair complexes in A549 cells treated with olaparib, IR and their combination. Furthermore, VRK1 reached its maximum effect on impairment of repair foci at lower doses of olaparib and IR.

### The combined DNA damage response to olaparib and IR is impaired by VRK1 depletion and is independent of ATM and p53

**Fig. 5:**
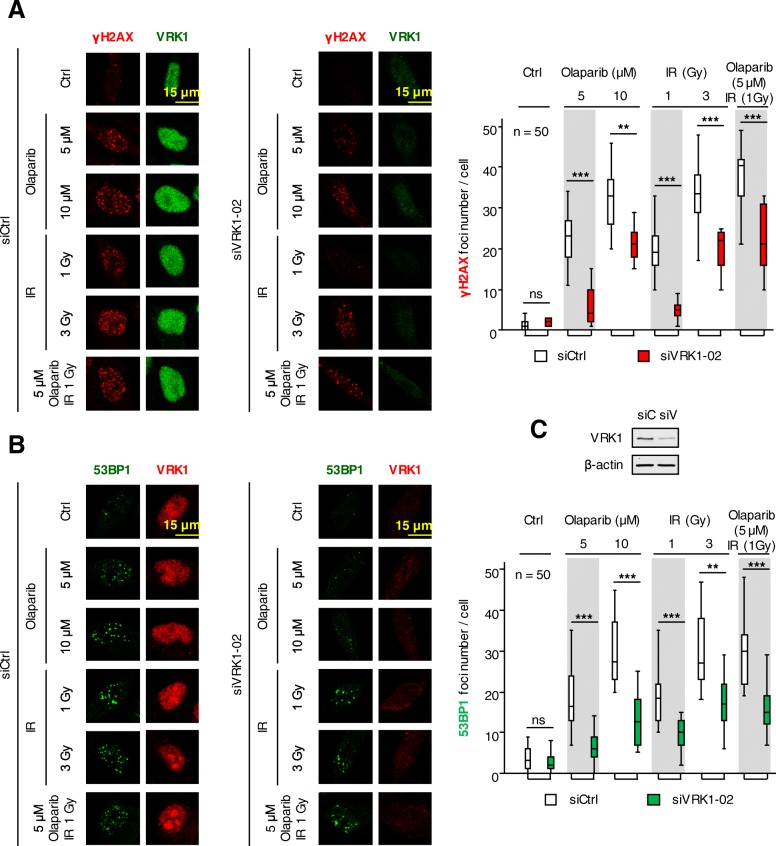
Quantification of the effect of VRK1 depletion on the formation of γH2AX and 53BP1 foci induced by olaparib, IR or their combination in HT144 (*ATM*−/−) cells. **a**. Effect of si-Control on HT144 cells treated with different doses of olaparib, IR or their combination on the formation of γH2AX foci. **b**. Effect of si-VRK1 on HT144 cells treated with different doses of olaparib, IR or their combination on the formation of 53BP1 foci in HT144 (*ATM*−/−) cells. **C.** Detection of VRK1 depletion in immunoblot. siC: siControl, siV: siVRK1–02. ns: not significant, ** *p* < 0.01, *** *p* < 0.001

VRK1 is an activator of p53 in response to DNA damage [52–54]. This kinase phosphorylates p53 in Thr18, which prevents its interaction with mdm2 and leads to its stabilization and accumulation [52, 55]. This activation of p53 mediated by VRK1 is reverted after DNA is repaired by an autoregulatory loop implicating autophagy [53]. Moreover, this autoregulation is defective in lung carcinomas with p53 mutations [53, 56]. Therefore, we tested whether depletion of VRK1 in HT144 (*ATM*−/−) and H1299 (*TP53*−/−) tumor cell lines could interfere with the response to olaparib. In ATM-defective cells, VRK1 knockdown resulted in a defective repair reaction and the loss of γH2AX and 53BP1 foci in response to olaparib and IR treatments (Fig. 5). Similar results were obtained in H1299 cells (Additional file 9: Figure S9).

### ATM inhibition also impairs DDR after treatment with olaparib

**Fig. 6:**
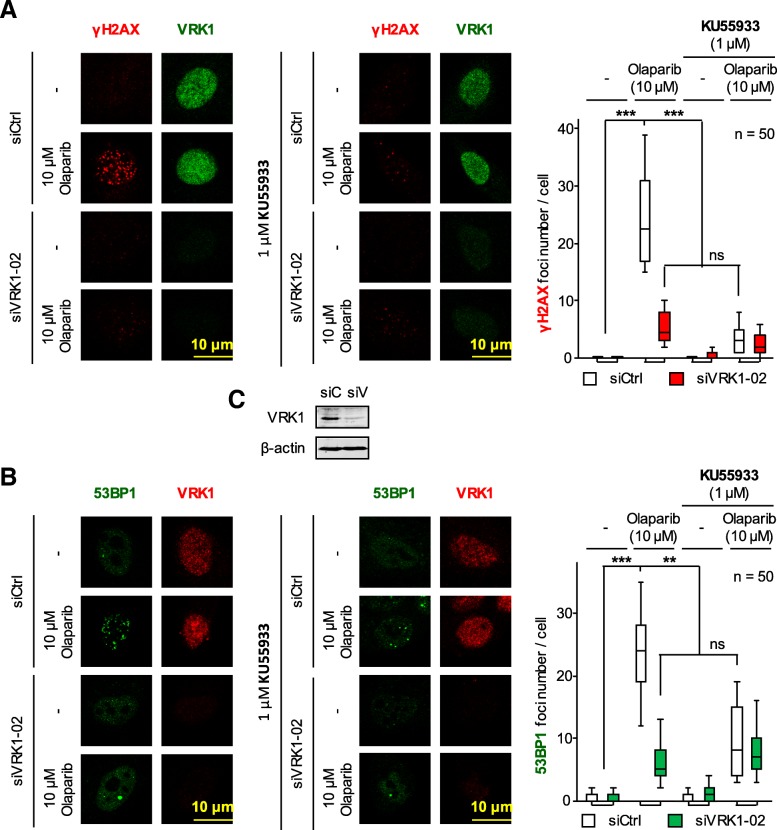
Effect of the ATM inhibitor KU55933 on the formation of γH2AX (**a**) and 53BP1 (**b**) foci induced by olaparib in A549 cells. **c**. Detection of VRK1 depletion in immunoblot. siC: siControl, siV: siVRK1–02. ns: not significant, ** *p* < 0.01, *** *p* < 0.001

VRK1 is upstream on ATM in DDR [20, 23, 24]. Therefore, we hypothesized that inhibition of ATM should cause a similar effect to VRK1 depletion, and impair the formation of γH2AX and 53BP1 foci induced by olaparib treatment. A549 cells were treated with the ATM inhibitor KU55933 as well as olaparib and the effect of their combination was determined. The addition of ATM inhibitor KU55933 to A549 cells (Fig. 6) also resulted in a reduction in the formation of foci induced by olaparib, which were also sensitive to VRK1 depletion. Thus, a combination of olaparib with a future inhibitor of VRK1 might be a suitable combination for tumors that are wild-type ATM.

### The initial H4K16 acetylation induced by olaparib is independent of ATM

**Fig. 7:**
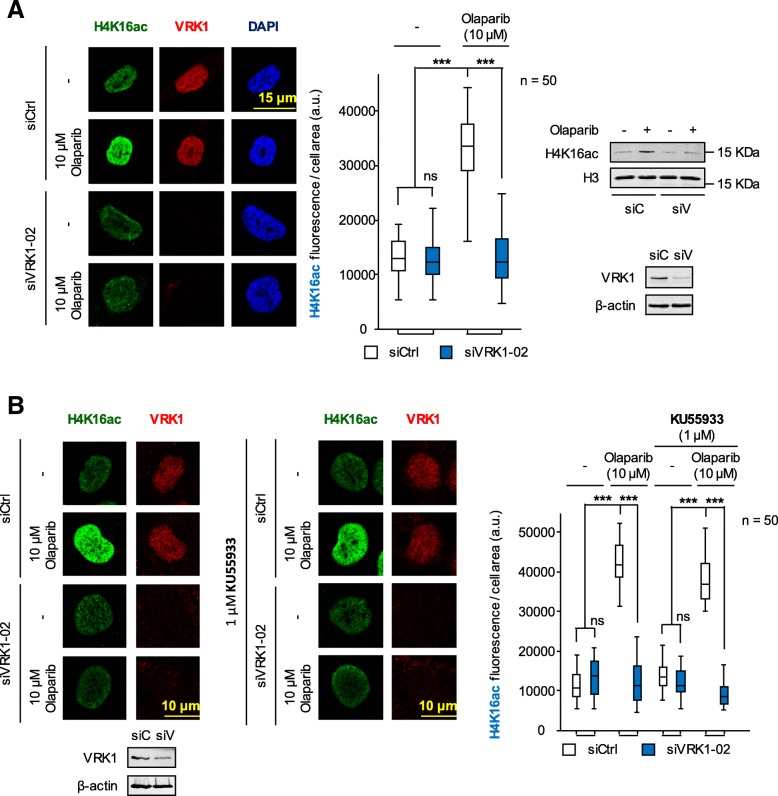
H4K16ac  induced by olaparib is independent of ATM. **a**. Effect of VRK1 depletion on H4K16 acetylation induced by olaparib treatment in HT144 (*ATM*−/−) cells. The field image used for quantification is shown in Additional file 10A: Figure S10A. **b.** Effect of VRK1 depletion on H4K16 acetylation induced by olaparib in A549 (*ATM*+/+) cells that were preincubated with the ATM inhibitor KU55933 for three hours before the addition of olaparib. The field image used for quantification is shown in Additional file 10B: Figure S10B. siC: siControl, siV: siVRK1–02. ns: not significant, *** *p* < 0.001

The initial response induced by olaparib, because of not repairing oxidized nucleotides, is a local chromatin relaxation that can be detected as an acetylation of histones, an effect that should be independent of ATM. To test this possibility, H4K16ac levels induced by olaparib were studied in cells that are ATM null, or in the presence of ATM inhibitor in *ATM* wild-type cells. Olaparib treatment induced an increase in the level of H4K16 ac in HT144 (*ATM*−/−) cells, which was sensitive to VRK1 depletion (Fig. 7a; field image in Additional file 10A: Figure S10A). To further confirm this effect, H4K16ac nuclear fluorescence was also determined in A549 (*ATM*+/+) cells that were preincubated with the ATM inhibitor KU55933 and treated with olaparib. In these conditions, H4K16ac was sensitive to VRK1 depletion as expected (Fig. 7B, left), and insensitive to the ATM inhibitor KU55933 (Fig. 7b, center; field image in Additional file 10B: Figure S10B). These observations indicated that the initial effect of olaparib on the level of H4K16 ac did not require ATM, and was dependent on VRK1.

## Discussion

Mutations in genes controlling genome stability are commonly associated with different types of hereditary cancer. These hereditary mutations increase the risk of cancer development and facilitate tumor progression. Somatic mutations in these genes can also occur during tumor progression if the mutation load does not interfere with cell viability. Therefore, tumors with defects in DNA repair pathways can become more sensitive to different forms of treatment, as well as more immunogenic [57, 58]. Moreover, the accumulation of excessive DNA damage in cells can reduce their viability and lead to their death. Because of that, induction of DNA damage by radiation or chemotherapeutic drugs that directly cause DNA damage is a common mechanism of action of cancer treatments. However, these therapeutic approaches frequently have a high toxicity and side effects leading to incomplete treatments. Therefore, improvement in cancer therapies requires the development of new strategies or combination therapies, which can reduce their toxicity by a decrease in doses employed.

**Fig. 8:**
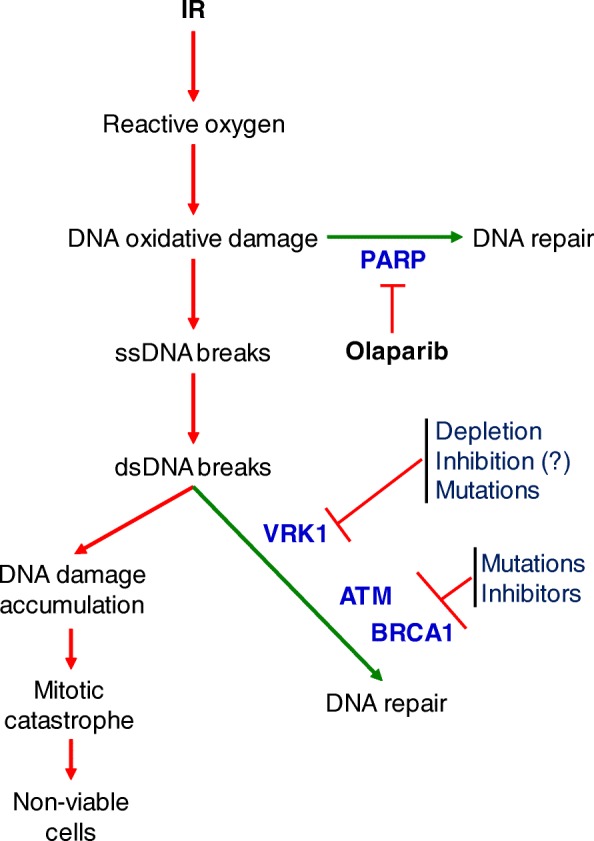
Diagram illustrating different steps in the response to DNA damage showing the cooperation of different treatments targeting specific DDR proteins, which can functionally mimic their mutation. The VRK1 inhibitor question mark means that they are not yet available because they have not been designed

Recently, the development of inhibitors that impair DNA repair processes and maximize the effects of common treatments is a new field. Olaparib has been shown to sensitize cells to these treatments in cells that have deficiencies in some DNA repair mechanisms and, for this reason, it has been used in breast and ovarian cancer with *BRCA1* mutations [17, 18], or in mantle cell lymphomas with alterations of the ATM pathway [15]. Thus, novel regulators of DNA repair mechanism are useful for development of new inhibitors to manage cancer with a reduced toxicity. In this context, the identification of VRK1 as a protein that can impair DDR may have therapeutic potential (Fig. 8). In fact, VRK1, because of the structural characteristics of its kinase domain, is the kinase with the smaller risk of cross inhibition with other Ser-Thr kinase inhibitors [59, 60]. The combined use of olaparib with VRK1 inhibitors can result in cancer treatments with less severe, or fewer, side effects and toxicity, and thus better tolerated. VRK1 depletion can function in cells lacking ATM or p53 [20, 23, 24, 26], which widens the number of tumor types where they can be used as an effective treatment. Furthermore, VRK1 has a dual role in DDR, since it also participates in the early chromatin relaxation as a consequence of DNA breaks and in individual steps of specific pathways, such as the phosphorylation of 53BP1 required for the assembly of 53BP1 foci [20]. In consequence, VRK1 knockdown clearly alters the DNA-repair process at different stages, from the early local chromatin relaxation associated with H4K16 acetylation to subsequent steps of DDR, such as H2AX, NBS1 and 53BP1 phosphorylation and accumulation, which are indicators of overall damage.

VRK1 plays a crucial role in cell proliferation and DNA repair processes, which, if inhibited, can lead to a reduction in tumor growth and an increase in its genetic instability that can be manipulated for therapeutic purposes, and perhaps also make the tumor more immunogenic. Furthermore, targeting VRK1 can also have additional effects on tumor progression. High levels of VRK1 has been associated as a bad prognostic indicator to several tumors such as breast [27, 61, 62], gliomas [28], colon [63], lung [47, 64], and hepatocarcinomas [30] among others. Mechanistically, these high VRK1 levels facilitate tumor cell proliferation [22, 45, 65], metastasis formation [66], and resistance to DNA damage [20, 26] mediated by the activation of repair process in chromatin [19] and by p53-mediated responses [52, 55, 56, 67, 68]. Therefore, the depletion of VRK1 will reduce proliferative potential and impair the response to DNA damage that will facilitate cell death. In a gene expression analysis of ninety-two human lung adenocarcinomas, which were compared to its matched controls, high VRK1 expression levels were detected in mitotic networks of lung adenocarcinomas, and its inhibition cooperated with PARP inhibitors to reduce tumor growth [47].

VRK1 plays a role in different steps of DDR [19] and its impartment can sensitize cells to treatments based on DNA damage by preventing the response. VRK1 knockdown facilitates the synthetic lethality by combination of olaparib and IR. The effect of VRK1 depletion on the cellular response to olaparib, by itself or in combination with IR, suggests that drugs targeting VRK1 might represent a novel therapeutic tool when they are developed. There is no inhibitor of VRK1, since its atypical catalytic site makes it insensitive to kinase inhibitors as demonstrated by interaction assays [59, 60] and kinase assays [69], but its development**,** considering its potential for a high specificity, might be an important therapeutic advance for use in combination therapies to minimize toxic drug doses, and limit the potential for resistance. Furthermore, it is important to note that the effect of VRK1 is independent of p53 or ATM and, thus, any potential new inhibitor targeting VRK1 could be used in cancers independently of their mutational status. Eventually, this would also permit alternative drug combinations that will have to be determined, and adjusted, for specific tumor types.

The dual effect of VRK1 depletion on reduction of cell cycle progression and defective DDR implicates that the absence of this kinase in arrested tumor stem cells can facilitate the accumulation of mutations. This effect could lead to a loss of tumor cell viability, or, alternatively, to a higher mutational load in tumor stem, or non-dividing, cells that could potentially make them more amenable to immunotherapy [70–73]. The combination of drugs targeting different participants in DDR pathways is a suitable form of cancer treatment that can result in reductions of drug or radiation doses and their toxicity. Moreover, the impairment of DDR by targeting new components of the pathway will facilitate accumulation of genetic damage in tumor cells, and make them more immunogenic and susceptible to new immunotherapeutic approaches.

## Conclusion

VRK1 depletion impairs the DNA damage response induced by olaparib, ionizing radiation and their combination, permitting a reduction in doses. Consequently, development of VRK1 inhibitors can be useful to treat tumor cells by impairing DNA repair processes, which would contribute to improve the efficacy of current treatments independently of the mutational status of p53 and ATM, and by facilitating accumulation of DNA damage that either lead to cell death, or make tumor cells more immunogenic.

## Additional files

Additional file 1: **Figure S1.** Effect of combinations of olaparib and ionizing radiation on the formation of γH2AX and 53BP1 foci in response to DNA damage in A549 cells. a. Effect of different doses of either olaparib or ionizing radiation on the formation of γH2AX and 53BP1 foci in response to DNA damage. b. Effect of combinations of olaparib and ionizing radiation on the formation of γH2AX and 53BP1 foci. The images (Fig. 1) show the detail of the subnuclear protein detected. The quantifications were performed using fifty cells from different fields of the experiments (usually between seven and ten were required). The images selected for presentation in the main Fig. 1 are indicated by boxes. (PDF 380 kb)

Additional file 2: **Figure S2**. Effect of combinations of olaparib and ionizing radiation on the formation of γH2AX and 53BP1 foci in response to DNA damage in H1299 (*TP53*−/−) cells. a. Effect of different doses of either olaparib or ionizing radiation on the formation of γH2AX and 53BP1 foci in response to DNA damage. b. Effect of combinations of olaparib and ionizing radiation on the formation of γH2AX and 53BP1 foci. c. Quantification of the effect of olaparib and IR by themselves or in combination on the number of γH2AX foci. d. Quantification of the effect of olaparib and IR, by themselves or in combination on the number of 53BP1 foci. ns: not significant, * *p* < 0.05, ** *p* < 0.01, *** *p* < 0.001. (PDF 350 kb)

Additional file 3: **Figure S3.** Effect of combinations of olaparib and ionizing radiation on the formation of γH2AX and 53BP1 foci in response to DNA damage in MDA-MB-231 breast cancer (triple negative) cells. a. Effect of different doses of either olaparib or ionizing radiation on the formation of γH2AX and 53BP1 foci in response to DNA damage. b. Effect of combinations of olaparib and ionizing radiation on the formation of γH2AX and 53BP1 foci. c. Quantification of the effect of olaparib and IR by themselves or in combination on the number of γH2AX foci. d. Quantification of the effect of olaparib and IR, by themselves or in combination on the number of 53BP1 foci. ns: not significant, *** *p* < 0.001. (PDF 312 kb)

Additional file 4: **Figure S4.** Effect of VRK1 depletion on the nuclear fluorescence associated to the acetylation of histone H4 in lysine 16 (H4K16ac ) induced by olaparib, IR or their combination in H1299 (*TP53*−/−) cells deprived (0.5%) of serum. The images show the detail of the subnuclear protein detected (Fig. 2). The quantifications were performed using fifty cells from different fields of the experiments (usually between seven and ten were required). The detail images selected for presentation in Fig. 2 are indicated by boxes. (PDF 735 kb)

Additional file 5: **Figure S5.** Effect of VRK1 depletion on the nuclear fluorescence associated to the acetylation of histone H4 in lysine 16 (H4K16 ac) induced by olaparib, IR or their combination in A549 (*TP53*+/+) cells deprived (0.5%) of serum. a left. Effect of siControl (siC) on A549 cells treated with different doses of olaparib, IR or their combination on nuclear H4K16ac fluorescence. a right. Effect of siVRK1 on A549 cells treated with different doses of olaparib, IR or their combination on the acetylation of histone H4 in lysine 16. b. Quantification of the effect of VRK1 depletion on the increase of nuclear H4K16ac fluorescence induced by DNA damage. c. The immunoblot shows the effect of VRK1 depletion on its protein level. ns: not significant. *** *p* < 0.001. (PDF 950 kb)

Additional file 6: **Figure S6.** Effect of VRK1 depletion on the nuclear NBS1 fluorescence induced by olaparib, IR or their combination in H1299 (*TP53*−/−) cells deprived (0.5%) of serum. The images show the detail of the subnuclear protein detected (Fig. 3). The quantifications were performed using fifty cells from different fields of the experiments (usually between seven and ten were required). The detail images selected for presentation in Fig. 3 are indicated by boxes. (PDF 596 kb)

Additional file 7: **Figure S7.** Effect of VRK1 depletion on nuclear NBS1 fluorescence induced by olaparib, IR or their combination in A549 (*TP53*+/+) cells deprived of serum (0.5%). A Left. Effect of siControl on A549 cells treated with different doses of olaparib, IR or their combination on the NBS1 fluorescence. A Right. Effect of si-VRK1 on A549 cells treated with different doses of olaparib, IR or their combination on the accumulation of NBS1 in nuclei. B**.** Quantification of the effect of VRK1 depletion on the increase of nuclear NBS1 fluorescence by aggregation of this protein induced by DNA damage. C. The immunoblot shows the effect of VRK1 depletion on its protein level. ns: not significant, ** *p* < 0.01, *** *p* < 0.001. (PDF 901 kb)

Additional file 8: **Figure S8**. Effect of VRK1 depletion on nuclear NBS1 fluorescence induced by olaparib, IR or their combination in HT144 (*ATM*−/−) cells deprived (0.5%) of serum. A left. Effect of siControl on HT144 cells treated with different doses of olaparib, IR or their combination on the NBS1 fluorescence. A right. Effect of siVRK1 on HT144 cells treated with different doses of olaparib, IR or their combination, on the accumulation of NBS1 in nuclei. B. Quantification of the effect of VRK1 depletion on the increase of nuclear NBS1 fluorescence by aggregation of this protein induced by DNA damage. c. The immunoblot shows the effect of VRK1 depletion on its protein level. ns: not significant. *** *p* < 0.001. (PDF 509 kb)

Additional file 9: **Figure S9.** Effect of VRK1 depletion on the formation of γH2AX and 53BP1 foci induced by olaparib, IR or their combination in H1299 (*TP53*−/−) cells. a. Effect of siControl on H1299 (*TP53*−/−) cells treated with different doses of olaparib, IR or their combination on the formation of γH2AX foci. b. Effect of siVRK1 on H1299 cells treated with different doses of olaparib, IR or their combination on the formation of 53BP1 foci in H1299 (*TP53*−/−) cells. c. Detection of VRK1 depletion in immunoblot. ns: not significant, * *p* < 0.05, ** *p* < 0.01, *** *p* < 0.001. (PDF 1248 kb)

Additional file 10: **Figure S10.** H4K16ac induced by olaparib is independent of ATM. A. Effect of VRK1 depletion on H4K16 acetylation induced by olaparib in HT144 (*ATM*−/−) cells. Field image used for quantification of H4K16ac . The number of cells counted is indicated in Fig. 7a. B. Effect of VRK1 depletion on H4K16 acetylation induced by olaparib in A549 (*ATM+/+*) cells that were preincubated with the ATM inhibitor KU55933 for three hours before the addition of olaparib. Field image used for quantification of H4K16ac . The number of cells counted is indicated in Fig. 7b. (PDF 414 kb)

## Abbreviations

ATM: Ataxia-telangiectasia mutated kinase
DDR: DNA-damage response
IR: Ionizing radiation
NBS1: Nijmegen breakage syndrome 1
PARP: Poly (ADP-Ribose) Polymerase
VRK1: Vaccinia-related kinase 1
