# Supplementary material for: Olaparib and ionizing radiation trigger a cooperative DNA-damage repair response that is impaired by depletion of the VRK1 chromatin kinase
Source: J Exp Clin Cancer Res. 2019 May 17;38:203. doi: 10.1186/s13046-019-1204-1 (PMC6525392; doi:10.1186/s13046-019-1204-1)
Supplement: Supplementary file 1 — Figure S1. Effect of combinations of olaparib and ionizing radiation on the formation of γH2AX and 53BP1 foci in response to DNA damage in A549 cells. a. Effect of different doses of either olaparib or ionizing radiation on the formation of γH2AX and 53BP1 foci in response to DNA damage. b. Effect of combinations of olaparib and ionizing radiation on the formation of γH2AX and 53BP1 foci. The images (Fig. 1) show the detail of the subnuclear protein detected. The quantifications were performed using fifty cells from different fields of the experiments (usually between seven and ten were required). The images selected for presentation in the main Fig. 1 are indicated by boxes. (PDF 380 kb) [file 13046_2019_1204_MOESM1_ESM.pdf]

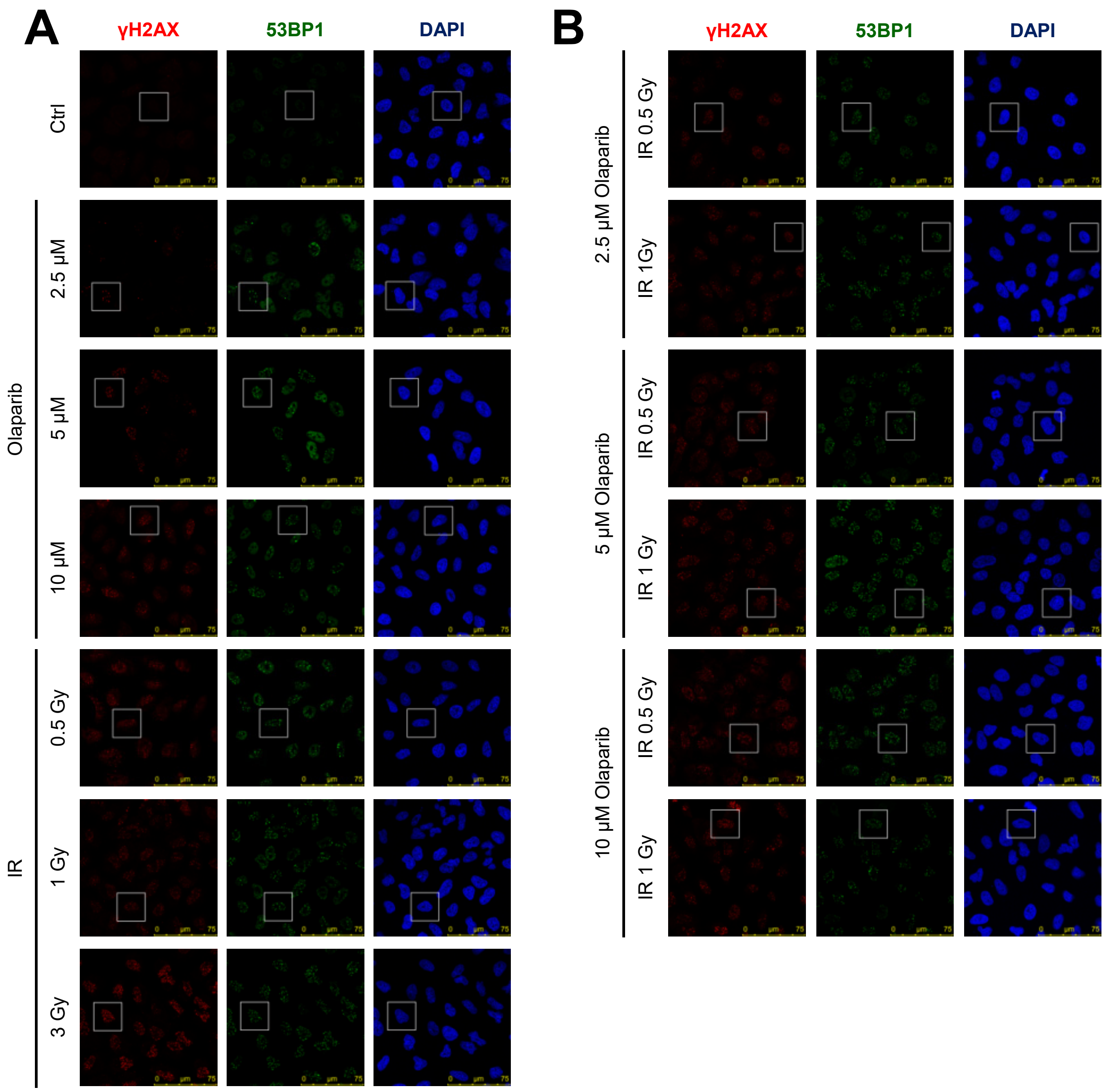

Figure S1. Effect of combinations of olaparib and ionizing radiation on the formation of γH2AX and 53BP1 foci in response to DNA damage in A549 cells. **A**. Effect of different doses of either olaparib or ionizing radiation on the formation of γH2AX and 53BP1 foci in response to DNA damage. **B**. Effect of combinations of olaparib and ionizing radiation on the formation of γH2AX and 53BP1 foci. The images (Fig. 1) show the detail of the subnuclear protein detected. The quantifications were performed using fifty cells from different fields of the experiments (usually between seven and ten were required). The images selected for presentation in the main Figure 1 are indicated by boxes.
