# Supplementary material for: Olaparib and ionizing radiation trigger a cooperative DNA-damage repair response that is impaired by depletion of the VRK1 chromatin kinase
Source: J Exp Clin Cancer Res. 2019 May 17;38:203. doi: 10.1186/s13046-019-1204-1 (PMC6525392; doi:10.1186/s13046-019-1204-1)
Supplement: Supplementary file 2 — Figure S2. Effect of combinations of olaparib and ionizing radiation on the formation of γH2AX and 53BP1 foci in response to DNA damage in H1299 (TP53−/−) cells. a. Effect of different doses of either olaparib or ionizing radiation on the formation of γH2AX and 53BP1 foci in response to DNA damage. b. Effect of combinations of olaparib and ionizing radiation on the formation of γH2AX and 53BP1 foci. c. Quantification of the effect of olaparib and IR by themselves or in combination on the number of γH2AX foci. d. Quantification of the effect of olaparib and IR, by themselves or in combination on the number of 53BP1 foci. ns: not significant, * p < 0.05, ** p < 0.01, *** p < 0.001. (PDF 350 kb) [file 13046_2019_1204_MOESM2_ESM.pdf]

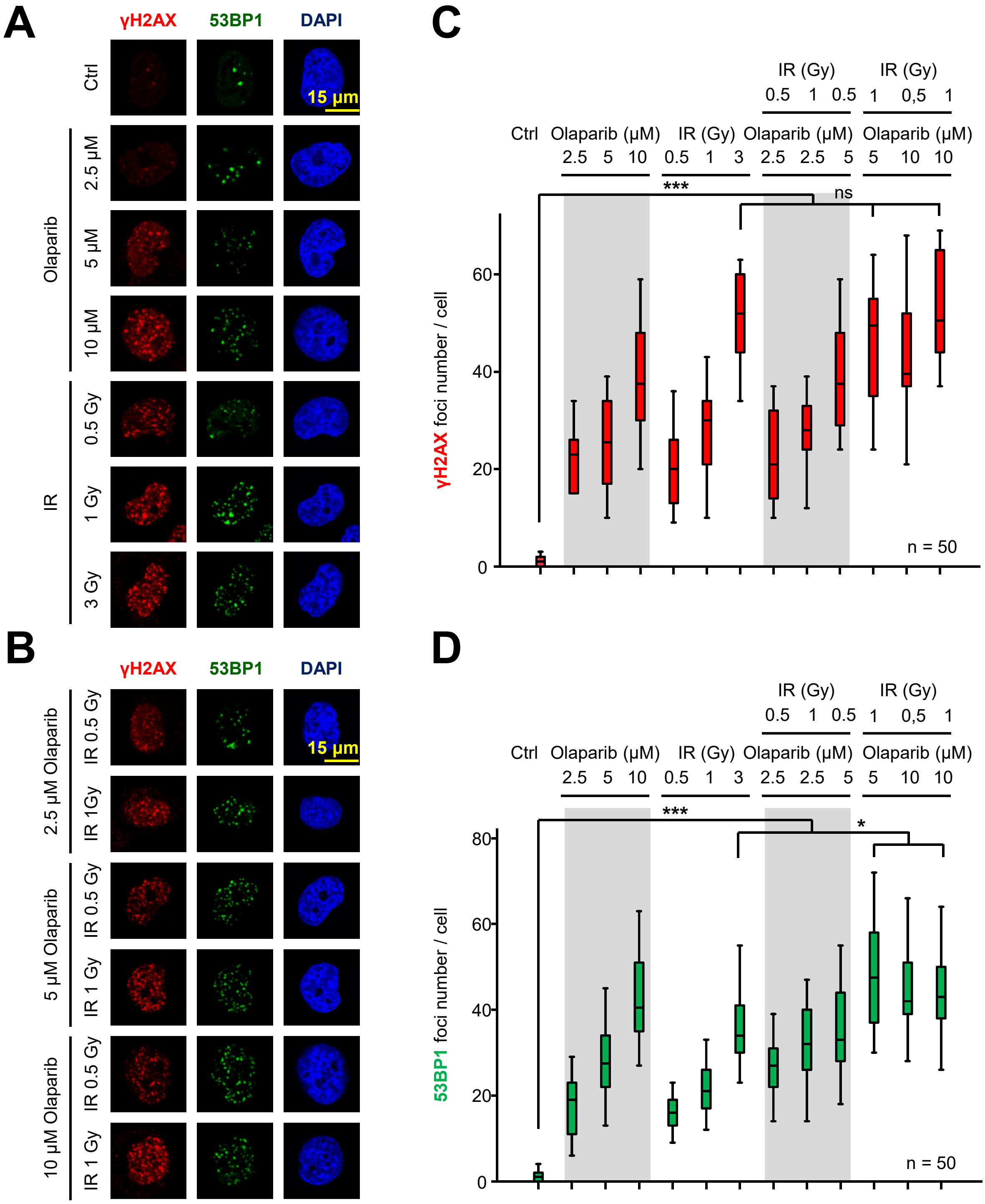


**Figure S2**. Effect of combinations of olaparib and ionizing radiation on the formation of γH2AX and 53BP1 foci in response to DNA damage in H1299 (*TP53*-/-) cells. **A**. Effect of different doses of either olaparib or ionizing radiation on the formation of γH2AX and 53BP1 foci in response to DNA damage. **B**. Effect of combinations of olaparib and ionizing radiation on the formation of γH2AX and 53BP1 foci. **C.** Quantification of the effect of olaparib and IR by themselves or in combination on the number of γH2AX foci. **D**. Quantification of the effect of olaparib and IR, by themselves or in combination on the number of 53BP1 foci. ns: not significant, * $p<0.05$, ** $p < 0.01$, *** $p < 0.001$.
