# Supplementary material for: Olaparib and ionizing radiation trigger a cooperative DNA-damage repair response that is impaired by depletion of the VRK1 chromatin kinase
Source: J Exp Clin Cancer Res. 2019 May 17;38:203. doi: 10.1186/s13046-019-1204-1 (PMC6525392; doi:10.1186/s13046-019-1204-1)
Supplement: Supplementary file 4 — Figure S4. Effect of VRK1 depletion on the nuclear fluorescence associated to the acetylation of histone H4 in lysine 16 (H4K16ac ) induced by olaparib, IR or their combination in H1299 (TP53−/−) cells deprived (0.5%) of serum. The images show the detail of the subnuclear protein detected (Fig. 2). The quantifications were performed using fifty cells from different fields of the experiments (usually between seven and ten were required). The detail images selected for presentation in Fig. 2 are indicated by boxes. (PDF 735 kb) [file 13046_2019_1204_MOESM4_ESM.pdf]

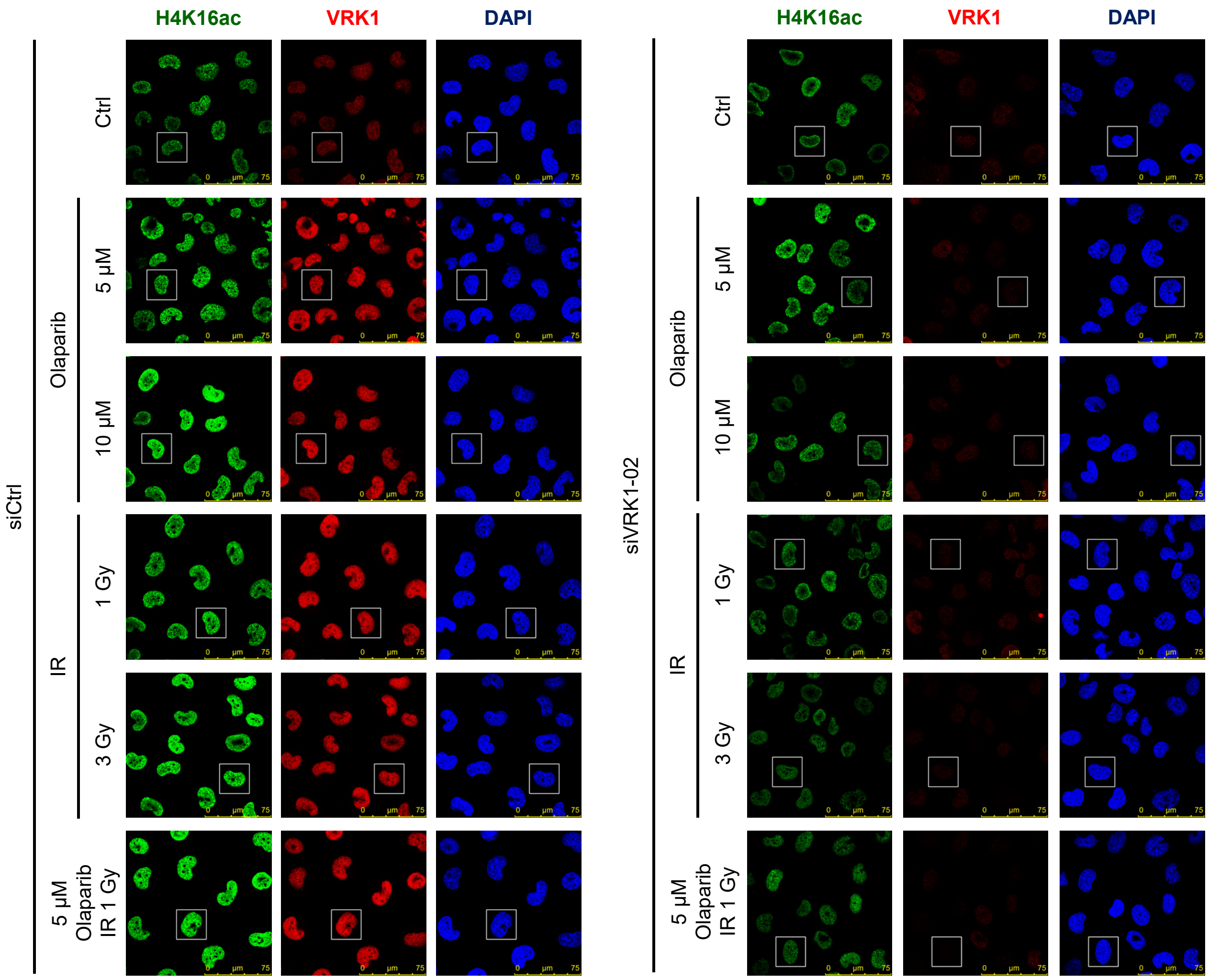

Figure S4. Effect of VRK1 depletion on the nuclear fluorescence associated to the acetylation of histone H4 in lysine 16 (H4K16ac) induced by olaparib, IR or their combination in H1299 (*TP53*-/-) cells deprived (0.5%) of serum. The images show the detail of the subnuclear protein detected (Fig.2). The quantifications were performed using fifty cells from different fields of the experiments (usually between seven and ten were required). The detail images selected for presentation in figure 2 are indicated by boxes.
