# Supplementary material for: Olaparib and ionizing radiation trigger a cooperative DNA-damage repair response that is impaired by depletion of the VRK1 chromatin kinase
Source: J Exp Clin Cancer Res. 2019 May 17;38:203. doi: 10.1186/s13046-019-1204-1 (PMC6525392; doi:10.1186/s13046-019-1204-1)
Supplement: Supplementary file 5 — Figure S5. Effect of VRK1 depletion on the nuclear fluorescence associated to the acetylation of histone H4 in lysine 16 (H4K16 ac) induced by olaparib, IR or their combination in A549 (TP53+/+) cells deprived (0.5%) of serum. a left. Effect of siControl (siC) on A549 cells treated with different doses of olaparib, IR or their combination on nuclear H4K16ac fluorescence. a right. Effect of siVRK1 on A549 cells treated with different doses of olaparib, IR or their combination on the acetylation of histone H4 in lysine 16. b. Quantification of the effect of VRK1 depletion on the increase of nuclear H4K16ac fluorescence induced by DNA damage. c. The immunoblot shows the effect of VRK1 depletion on its protein level. ns: not significant. *** p < 0.001. (PDF 950 kb) [file 13046_2019_1204_MOESM5_ESM.pdf]

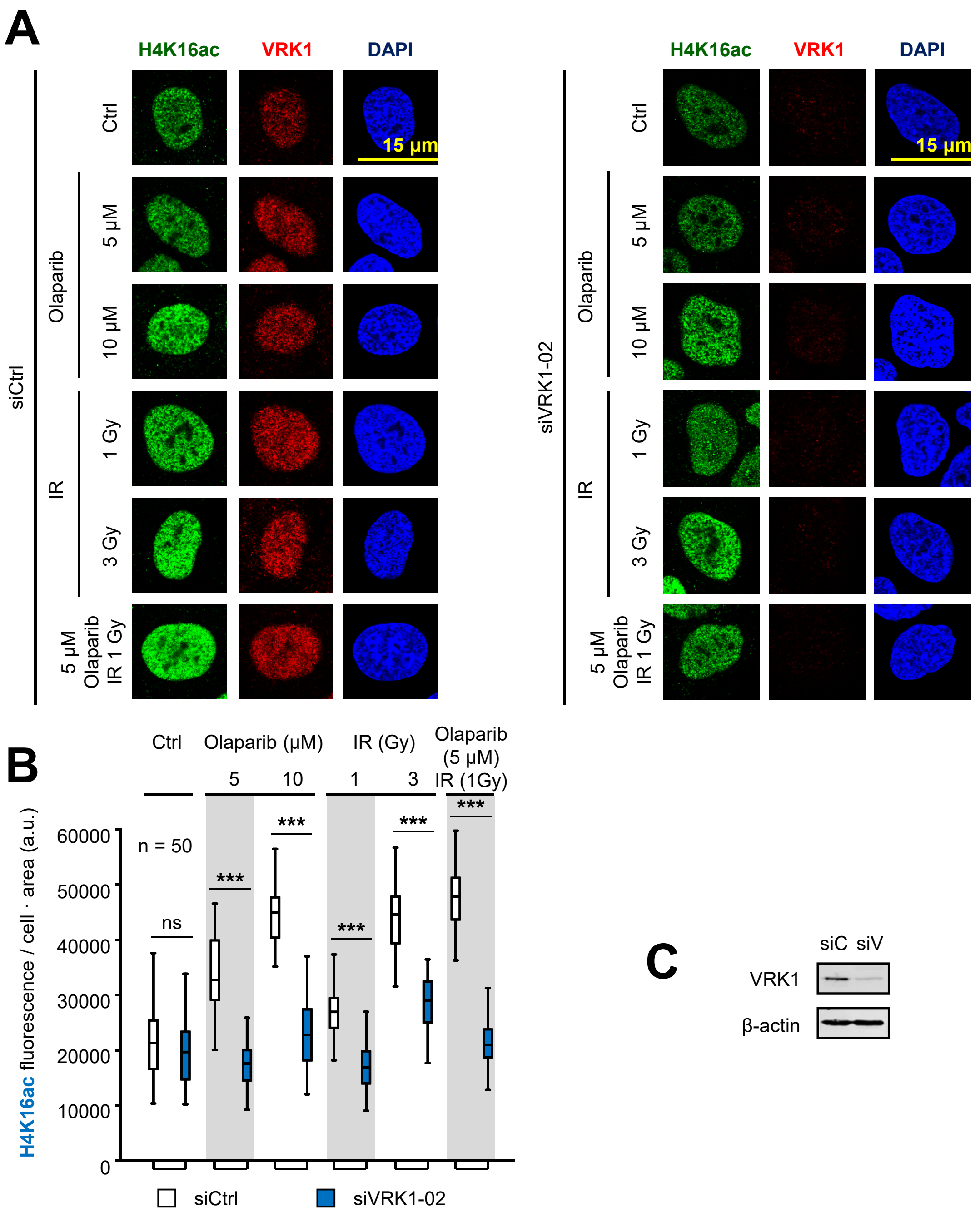

Figure S5. Effect of VRK1 depletion on the nuclear fluorescence associated to the acetylation of histone H4 in lysine 16 (H4K16ac) induced by olaparib, IR or their combination in A549 (*TP53*+/+) cells deprived (0.5%) of serum. **A left.** Effect of siControl (siC) on A549 cells treated with different doses of olaparib, IR or their combination on nuclear H4K16ac fluorescence. **A right.** Effect of siVRK1 on A549 cells treated with different doses of olaparib, IR or their combination on the acetylation of histone H4 in lysine 16. **B.** Quantification of the effect of VRK1 depletion on the increase of nuclear H4K16ac fluorescence induced by DNA damage. **C.** The immunoblot shows the effect of VRK1 depletion on its protein level. ns: not significant. *** $p < 0.001$.
