# Supplementary material for: Olaparib and ionizing radiation trigger a cooperative DNA-damage repair response that is impaired by depletion of the VRK1 chromatin kinase
Source: J Exp Clin Cancer Res. 2019 May 17;38:203. doi: 10.1186/s13046-019-1204-1 (PMC6525392; doi:10.1186/s13046-019-1204-1)
Supplement: Supplementary file 7 — Figure S7. Effect of VRK1 depletion on nuclear NBS1 fluorescence induced by olaparib, IR or their combination in A549 (TP53+/+) cells deprived of serum (0.5%). A Left. Effect of siControl on A549 cells treated with different doses of olaparib, IR or their combination on the NBS1 fluorescence. A Right. Effect of si-VRK1 on A549 cells treated with different doses of olaparib, IR or their combination on the accumulation of NBS1 in nuclei. B. Quantification of the effect of VRK1 depletion on the increase of nuclear NBS1 fluorescence by aggregation of this protein induced by DNA damage. C. The immunoblot shows the effect of VRK1 depletion on its protein level. ns: not significant, ** p < 0.01, *** p < 0.001. (PDF 901 kb) [file 13046_2019_1204_MOESM7_ESM.pdf]

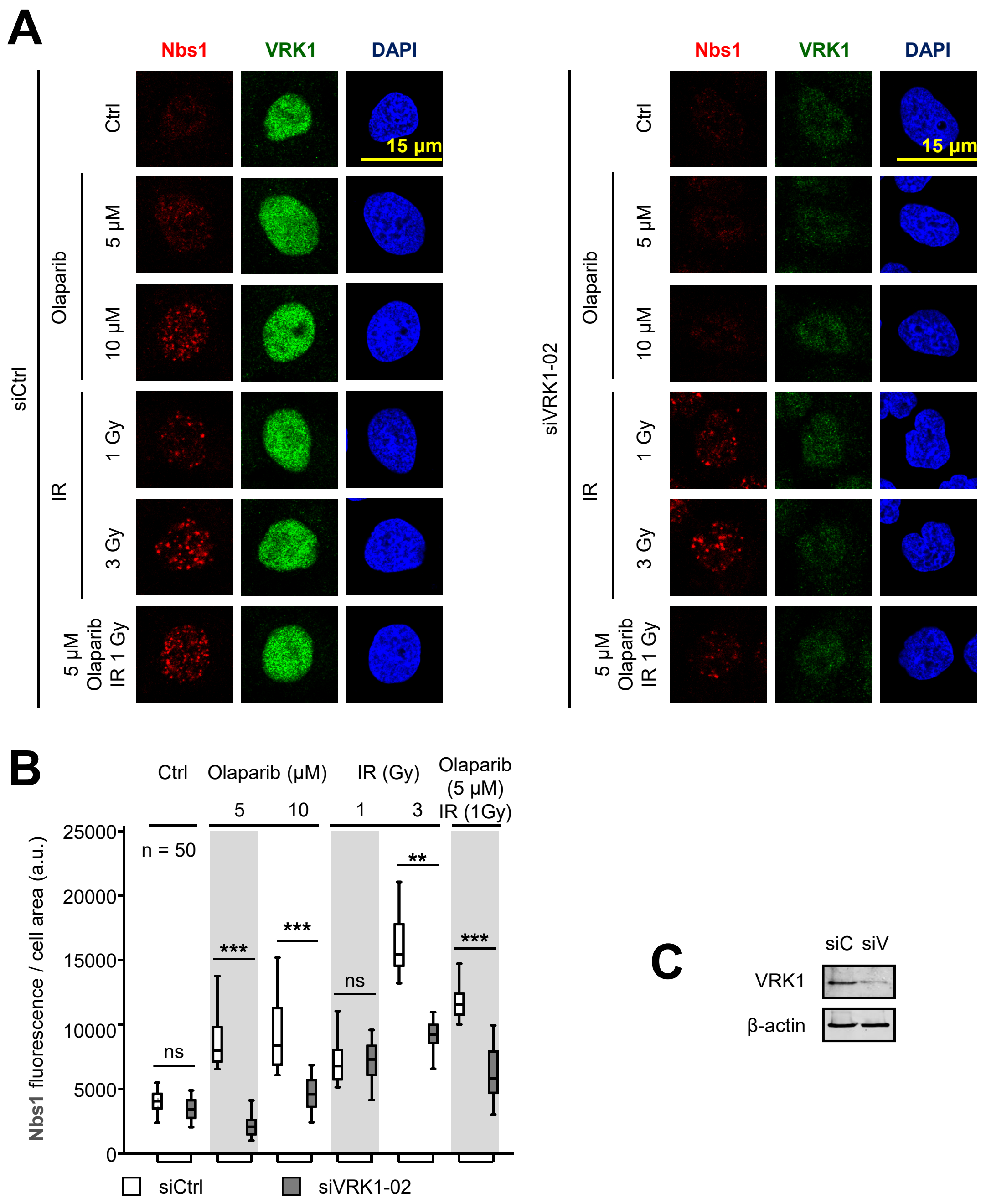

Figure S7. Effect of VRK1 depletion on nuclear NBS1 fluorescence induced by olaparib, IR or their combination in A549 (*TP53*+/+) cells deprived (0.5 %) of serum. **A left.** Effect of siControl on A549 cells treated with different doses of olaparib, IR or their combination on the NBS1 fluorescence. **A right.** Effect of si-VRK1 on A549 cells treated with different doses of olaparib, IR or their combination on the accumulation of NBS1 in nuclei. **B.** Quantification of the effect of VRK1 depletion on the increase of nuclear NBS1 fluorescence by aggregation of this protein induced by DNA damage. **C.** The immunoblot shows the effect of VRK1 depletion on its protein level. ns: not significant, ** $p < 0.01$, *** $p < 0.001$.
