# Supplementary material for: Olaparib and ionizing radiation trigger a cooperative DNA-damage repair response that is impaired by depletion of the VRK1 chromatin kinase
Source: J Exp Clin Cancer Res. 2019 May 17;38:203. doi: 10.1186/s13046-019-1204-1 (PMC6525392; doi:10.1186/s13046-019-1204-1)
Supplement: Supplementary file 8 — Figure S8. Effect of VRK1 depletion on nuclear NBS1 fluorescence induced by olaparib, IR or their combination in HT144 (ATM−/−) cells deprived (0.5%) of serum. A left. Effect of siControl on HT144 cells treated with different doses of olaparib, IR or their combination on the NBS1 fluorescence. A right. Effect of siVRK1 on HT144 cells treated with different doses of olaparib, IR or their combination, on the accumulation of NBS1 in nuclei. B. Quantification of the effect of VRK1 depletion on the increase of nuclear NBS1 fluorescence by aggregation of this protein induced by DNA damage. c. The immunoblot shows the effect of VRK1 depletion on its protein level. ns: not significant. *** p < 0.001. (PDF 509 kb) [file 13046_2019_1204_MOESM8_ESM.pdf]

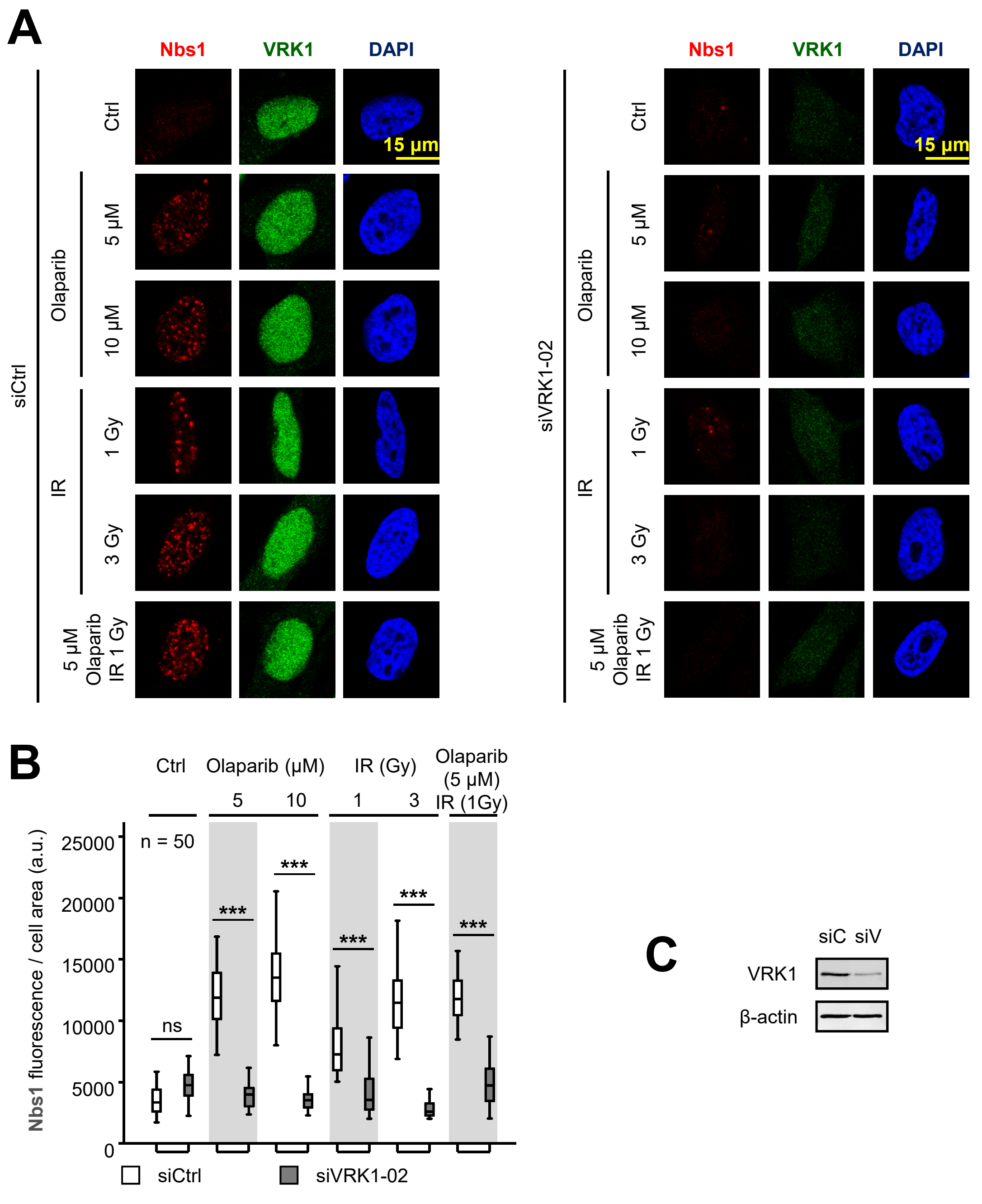

Figure S8. Effect of VRK1 depletion on nuclear NBS1 fluorescence induced by olaparib, IR or their combination in HT144 (*ATM*-/-) cells deprived (0.5 %) of serum. **A left.** Effect of siControl on HT144 cells treated with different doses of olaparib, IR or their combination on the NBS1 fluorescence. **A right.** Effect of siVRK1 on HT144 cells treated with different doses of olaparib, IR or their combination, on the accumulation of NBS1 in nuclei. **B.** Quantification of the effect of VRK1 depletion on the increase of nuclear NBS1 fluorescence by aggregation of this protein induced by DNA damage. **C.** The immunoblot shows the effect of VRK1 depletion on its protein level. ns: not significant. *** $p < 0.001$.
