# Supplementary material for: Olaparib and ionizing radiation trigger a cooperative DNA-damage repair response that is impaired by depletion of the VRK1 chromatin kinase
Source: J Exp Clin Cancer Res. 2019 May 17;38:203. doi: 10.1186/s13046-019-1204-1 (PMC6525392; doi:10.1186/s13046-019-1204-1)
Supplement: Supplementary file 9 — Figure S9. Effect of VRK1 depletion on the formation of γH2AX and 53BP1 foci induced by olaparib, IR or their combination in H1299 (TP53−/−) cells. a. Effect of siControl on H1299 (TP53−/−) cells treated with different doses of olaparib, IR or their combination on the formation of γH2AX foci. b. Effect of siVRK1 on H1299 cells treated with different doses of olaparib, IR or their combination on the formation of 53BP1 foci in H1299 (TP53−/−) cells. c. Detection of VRK1 depletion in immunoblot. ns: not significant, * p < 0.05, ** p < 0.01, *** p < 0.001. (PDF 1248 kb) [file 13046_2019_1204_MOESM9_ESM.pdf]

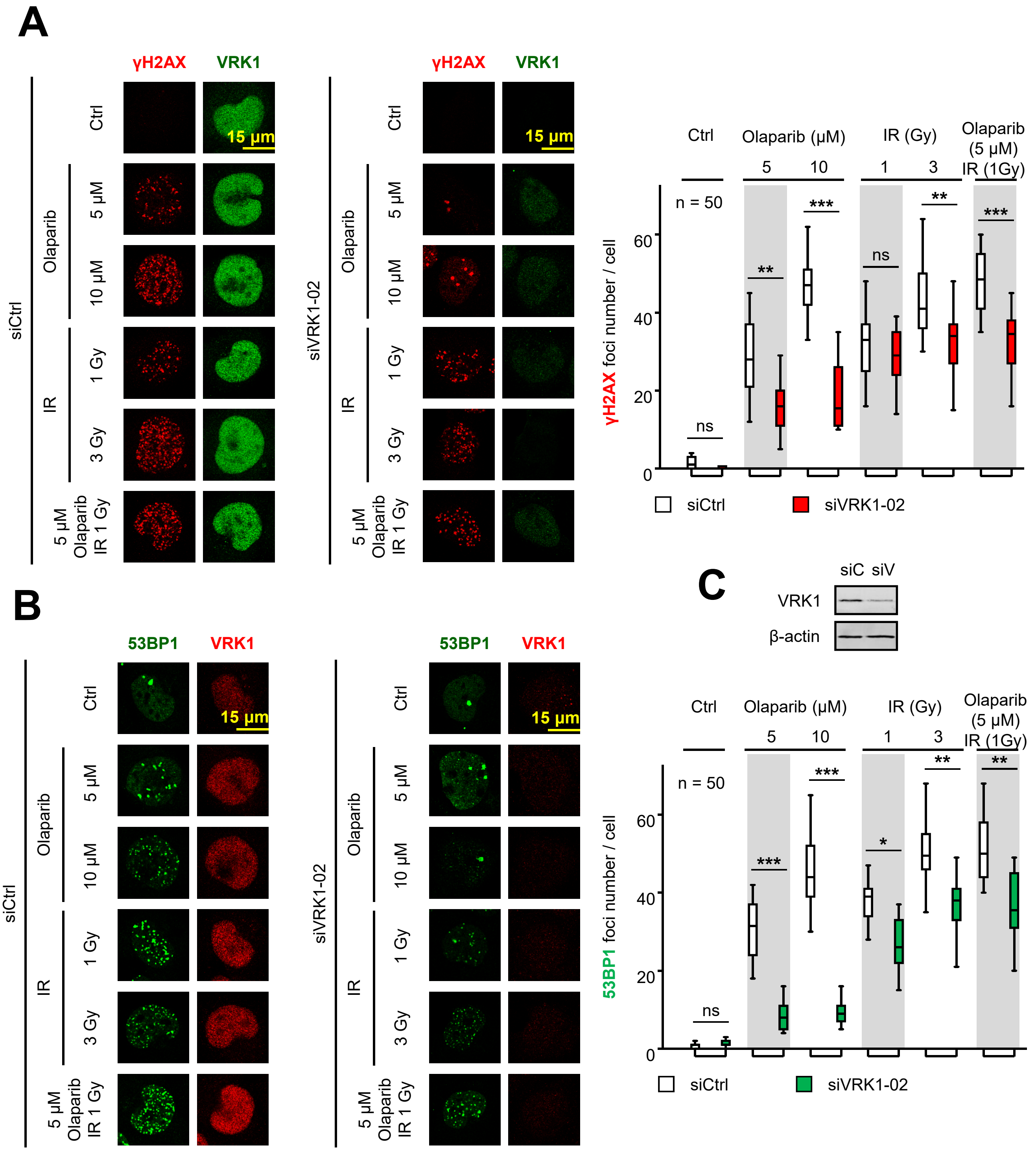

Figure S9. Effect of VRK1 depletion on the formation of γH2AX and 53BP1 foci induced by olaparib, IR or their combination in H1299 (p53-/-) cells. **A.** Effect of siControl on H1299 (*TP53*-/-) cells treated with different doses of olaparib, IR or their combination on the formation of γH2AX foci. **B.** Effect of siVRK1 on H1299 cells treated with different doses of olaparib, IR or their combination on the formation of 53BP1 foci in H1299 (*TP53*-/-) cells. **C.** Detection of VRK1 depletion in immunoblot. ns: not significant, * $p < 0.05$, ** $p < 0.01$, *** $p < 0.001$.
