# Supplementary material for: Olaparib and ionizing radiation trigger a cooperative DNA-damage repair response that is impaired by depletion of the VRK1 chromatin kinase
Source: J Exp Clin Cancer Res. 2019 May 17;38:203. doi: 10.1186/s13046-019-1204-1 (PMC6525392; doi:10.1186/s13046-019-1204-1)
Supplement: Supplementary file 10 — Figure S10. H4K16ac induced by olaparib is independent of ATM. A. Effect of VRK1 depletion on H4K16 acetylation induced by olaparib in HT144 (ATM−/−) cells. Field image used for quantification of H4K16ac . The number of cells counted is indicated in Fig. 7a. B. Effect of VRK1 depletion on H4K16 acetylation induced by olaparib in A549 (ATM+/+) cells that were preincubated with the ATM inhibitor KU55933 for three hours before the addition of olaparib. Field image used for quantification of H4K16ac . The number of cells counted is indicated in Fig. 7b. (PDF 414 kb) [file 13046_2019_1204_MOESM10_ESM.pdf]

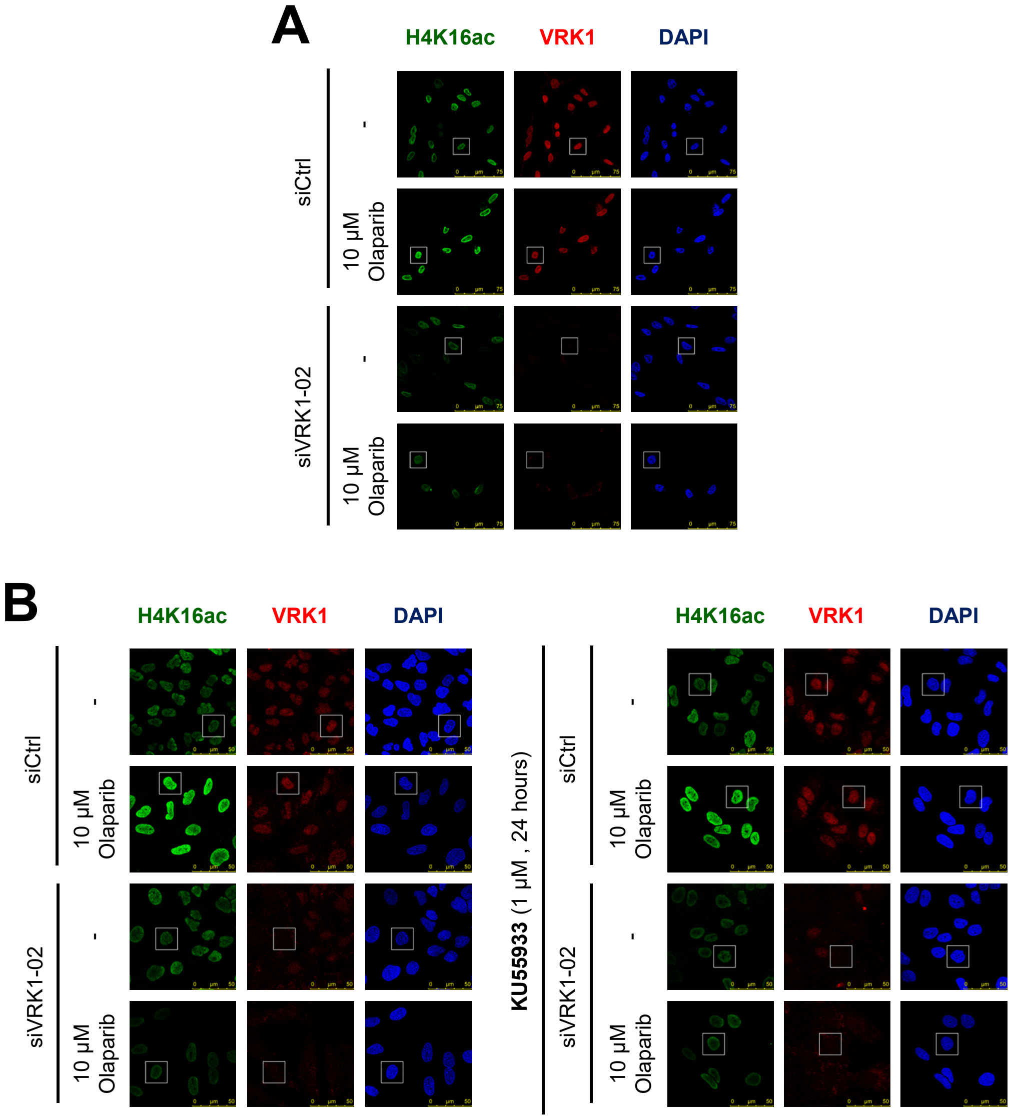

Figure S10. H4K16ac induced by olaparib is independent of ATM. **A**. Effect of VRK1 depletion on H4K16 acetylation induced by olaparib in HT144 (*ATM*-/-) cells. Field image used for quantification of H4K16ac. The number of cells counted is indicated in Figure 7A. **B.** Effect of VRK1 depletion on H4K16 acetylation induced by olaparib in A549 (*ATM*+/+) cells that were preincubated with the ATM inhibitor KU55933 for three hours before the addition of olaparib. Field image used for quantification of H4K16ac.The number of cells counted is indicated in Figure 7B.
